# Obinutuzumab plus chlorambucil versus ibrutinib in previously untreated chronic lymphocytic leukemia patients without TP53 disruptions: A real-life CLL campus study

**DOI:** 10.3389/fonc.2022.1033413

**Published:** 2022-11-21

**Authors:** Andrea Visentin, Francesca Romana Mauro, Gioachino Catania, Alberto Fresa, Candida Vitale, Alessandro Sanna, Veronica Mattiello, Francesca Cibien, Paolo Sportoletti, Massimo Gentile, Gian Matteo Rigolin, Francesca Maria Quaglia, Roberta Murru, Alessandro Gozzetti, Stefano Molica, Monia Marchetti, Stefano Pravato, Francesco Angotzi, Alessandro Cellini, Lydia Scarfò, Gianluigi Reda, Marta Coscia, Luca Laurenti, Paolo Ghia, Robin Foà, Antonio Cuneo, Livio Trentin

**Affiliations:** ^1^ Hematology and Clinical Immunology Unit, Department of Medicine, University of Padua, Padova, Italy; ^2^ Veneto Institute of Molecular Medicine, Padua, Italy; ^3^ Hematology, Department of Translational and Precision Medicine, “Sapienza” University, Rome, Italy; ^4^ Division of Hematology, Hospital Saints (A. O. SS) Antonio e Biagio and Cesare Arrigo, Alessandria, Italy; ^5^ Hematology Institute, Fondazione Policlinico Universitario Agostino Gemelli IRCSS, Rome, Italy; ^6^ Department of Molecular Biotechnology and health Sciences, University of Torino and Division of Hematology, University Hospital (A.O.U.) Città della Salute e della Scienza di Torino, Torino, Italy; ^7^ Hematology Unit, Careggi Hospital, Florence, Italy; ^8^ Hematology Unit, Fondazione IRCCS Ca’ Granda Ospedale Maggiore, University of Milan, Milan, Italy; ^9^ Hematology Unit, Ca’ Foncello Hospital, Treviso, Italy; ^10^ Hematology and Clinical Immunology Unit, University of Perugia, Perugia, Italy; ^11^ Hematology Section, Cosenza Hospital, Cosenza, Italy; ^12^ Hematology Section, Department of Medical Sciences, Azienda Ospedaliera-Universitaria, Arcispedale S. Anna, University of Ferrara, Ferrara, Italy; ^13^ Department of Medicine, Section of Hematology, University of Verona and Azienda Ospedaliera Universitaria Integrata, Verona, Italy; ^14^ Hematology and Stem Cell Transplantation Unit, Ospedale A. Businco ARNAS “G. Brotzu”, Cagliari, Italy; ^15^ Hematology Unit, University of Siena, Siena, Italy; ^16^ Department Hematology-Oncology, Azienda Ospedaliera Pugliese-Ciaccio, Catanzaro, Italy; ^17^ Strategic Program on CLL, University Health and Science “San Raffaele”, Milan, Italy

**Keywords:** obinutizumab, ibrutinib, treatment-naive, MRD, economic impact

## Abstract

One of the main issues in the treatment of patients with chronic lymphocytic leukemia (CLL) deals with the choice between continuous or fixed-duration therapy. Continuous ibrutinib (IB), the first-in-class BTK inhibitor, and obinutuzumab-chlorambucil (G-CHL) are commonly used therapies for elderly and/or comorbid patients. No head-to-head comparison has been carried out. Within the Italian campus CLL network, we performed a retrospective study on CLL patients without TP53 disruption treated with IB or G-CHL as first-line therapy. Patients in the G-CHL arm had a higher CIRS score and the worst renal function. The overall response rates between the G-CHL and IB arms were similar, but more complete remissions (CRs) were achieved with G-CHL (*p* = 0.0029). After a median follow-up of 30 months, the progression-free survival (PFS, *p* = 0.0061) and time to next treatment (TTNT, *p* = 0.0043), but not overall survival (OS, *p* = 0.6642), were better with IB than with G-CHL. Similar results were found after propensity score matching and multivariate analysis. While PFS and TTNT were longer with IB than with G-CHL in IGHV unmutated patients (*p* = 0.0190 and 0.0137), they were superimposable for IGHV mutated patients (*p* = 0.1900 and 0.1380). In the G-CHL arm, the depth of response (79% *vs*. 68% *vs*. 38% for CR, PR and SD/PD; *p* < 0.0001) and measurable residual disease (MRD) influenced PFS (78% *vs*. 53% for undetectable MRD *vs*. detectable MRD, *p* = 0.0203). Hematological toxicities were common in the G-CHL arm, while IB was associated with higher costs. Although continuous IB provides better disease control in CLL, IGHV mutated patients and those achieving an undetectable MRD show a marked clinical and economic benefit from a fixed-duration obinutuzumab-based treatment.

## Introduction

The treatment landscape of chronic lymphocytic leukemia (CLL) has significantly changed in the last few years thanks to the discovery of targeted drugs directed against pivotal kinases, such as BTK [ibrutinib (IB), acalabrutinib, zanubrutinib, and pirtobrutinib] or PI3K (idelalisib and duvelisib), anti-apoptotic protein, such as BCL2 (venetoclax), and new monoclonal antibodies targeting CD19 (tafasitamab) or CD20 (1–3). Among the latter, obinutuzumab (G), a glycoengineered type II humanized anti-CD20 monoclonal antibody, displays increased direct cell death, B-cell depletion, FcγRIIIa binding, and antibody-dependent cell-mediated cytotoxicity, and it has a lower capacity to re-localize CD20 into lipid rafts upon binding and to decrease complement-dependent cytotoxicity (4, 5).

The current frontline therapy of CLL patients can be either a continuous BTK inhibitor or a fixed-duration G-based therapy (1). Choosing between the two approaches remains a challenge, since a continuous treatment might offer better disease control for some aggressive subsets of patients, balanced however by an increased rate of long-term adverse events (AEs) and costs for the health system (6–9). On the other hand, a fixed G-based therapy is administered for a short period, allowing the achievement of deep remission, which is likely to be less prone to the development of resistant clones, but requires an outpatient clinic admission (10–12).

There is no head-to-head comparison between IB and G-chlorambucil (G-CHL) both in clinical trials and in real-life studies. A cross-trial comparison between Resonate-II and CLL11 suggests that overall IB seems to be better than G-CHL (13) in terms of survival analysis and safety profile during the first 6 months of treatment (grade 3 events, 50% *vs*. 71%). Furthermore, there are only a few retrospective studies that have assessed the efficacy of G-CHL and measurable residual disease (MRD) in the real-life setting (14–18).

In this study, we performed a retrospective study within the Italian Campus CLL network comparing the efficacy, MRD rates, safety, and economic cost of G-CHL *vs*. IB in treatment-naive CLL patients. We found that IB provides better disease control in most cases, but those patients who were IGHV mutated (M-IGHV) patients and who achieved an undetectable MRD (uMRD) showed a sustained clinical and economic benefit from a fixed-duration G-CHL-based therapy.

## Methods

### Study design

This is a retrospective study aimed at collecting and analyzing data of CLL patients treated outside of clinical trials with frontline IB or G-CHL from their reimbursement in Italy up to December 2021. Inclusion criteria were (i) diagnosis of CLL and the need for treatment according to the iwCLL 2018 guidelines (19) and (ii) patients unfit for fludarabine-based therapy (as evaluated by the treating physician). Exclusion criteria were (i) unable to sign the informed consent, (ii) relapsed/refractory patients, and (iii) ECOG >3.

Patients received IB 420 mg daily until progression or unacceptable toxicity, while G was administered at 100 mg on day 1, 900 mg on day 2, and 1,000 mg on days 8 and 15 of the first cycle, then at 1,000 mg of day 1 of cycles 2–6. CHL was used at the dose of 0.5 mg/kg every 2 weeks or according to local policies.

Efficacy and survival analyses were focused in patients without TP53 abnormalities (including FISH 17p13 deletion and/or TP53 mutation). The primary endpoint was progression-free survival (PFS) with G-CHL *vs*. IB. Secondary endpoints were overall response rate (ORR), which included complete remission (CR) and partial remission with/without lymphocytosis (PR-L and PR), time to next treatment (TTNT), overall survival (OS), subgroup analyses, locally performed flow cytometry to assess measurable residual disease (MRD), AEs, and economic impact of treatments.

In order to compare the costs of the drugs, we used the ex-factory prices in Italy in 2021: €16.47 for CHL (os, 2 mg each pill, 25 pills in each box), €2,828.63 for G (ev, 1 bottle, 1,000 mg), and €7,299.59 for IB (os, 140 mg each pill, 90 pills in each box). Costs of outpatient visits (€14.50), emergency room accesses, and days of hospitalization (€530/day) were counted based on the regional prices of reimbursement. Costs of other concomitant therapies were not included.

### Biological markers and MRD analysis

Cytogenetics by FISH (20, 21), TP53 mutation (22), and IGHV mutational status (23, 24) were performed in all recruited patients in local accredited laboratories, and their protocols are summarized in the supplementary materials. An IGHV gene sequence homology ≥98% was considered as unmutated (U-IGHV), as opposed to mutated (M-IGHV) (25). For MRD assessed by flow cytometry, mononuclear cells were marked according to the ERIC protocol (26) or its update. Briefly, 1,000,000–2,000,000 events were acquired for each sample and analyzed by Infinicyt™. MRD was considered undetectable when <10^−4^ (uMRD), as opposed to detectable MRD (dMRD) (27). MRD was not performed in patients with progressive disease (PD) at response assessment.

### Statistical analysis

Categorical variables were compared by the Chi-square test or the Fisher exact test, when appropriate. Continuous variables were compared using the Mann–Whitney test. PFS was calculated as start time of treatment to disease relapse or death (event) or last known follow-up (censured). TTNT was calculated according to the start time of G-CHL or IB to the beginning of a new line of therapy (event) or last known follow-up (censured). OS was calculated starting from the start of CLL treatment to death for any cause or last known follow-up. Survival analyses were performed by the Kaplan–Meier method, and the Log-rank test was used to compare survival curves between groups. The prognostic impact for the outcome variables was investigated by univariate and multiple Cox regression analysis. In Cox models, data were expressed as hazard ratios (HRs) and 95% confidence intervals (CIs). All covariates as well as all variables significantly unbalanced between the two study arms were jointly introduced into the same multiple Cox regression model (6). A propensity score matching analysis (1:1) with and without resampling was also carried out with a 0.2 caliper width. A *p*-value < 0.05 was considered as statistically significant. Correction for multiple comparison was also applied when indicated.

## Results

### Patients

**Figure 1:**
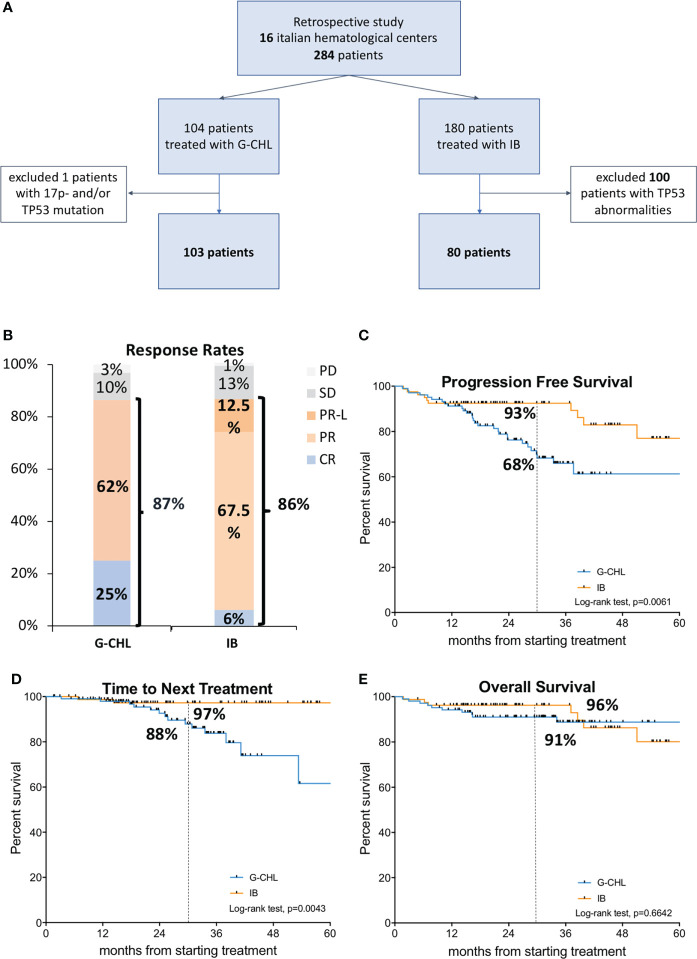
Study design and treatment efficacy. In the upper panel **(A)**, the consort plot of the study is shown. Among the 284 patients recruited within 16 Italian hematological centers, 101 were excluded due to the presence of TP53 abnormalities, including deletion of 17p13 (17p-) and/or TP53 mutation. In the middle panel, on the left **(B)**, the response rates plot for G-CHL (*n* = 103) and IB treatments are shown, and on the right **(C)**, the Kaplan–Meier curve of progression-free survival of G-CHL (*n* = 103) *vs*. IB (*n* = 80) is displayed. In the lower panel, on the left **(D)**, the Kaplan–Meier curves of time to next treatment is shown, and on the right **(E)**, that of overall survival of G-CHL (*n* = 103) *vs*. IB. (*n* = 80) is displayed.

We collected data of 284 patients from 16 Italian hematological centers within the Italian CLL campus network; 104 patients received G-CHL as frontline treatment and 180 patients were treated with IB. As shown in the consort plot, we excluded 101 patients due to the presence TP53 abnormalities: 1 subject in the G-CHL arm, and 100 patients in the IB arm [the latter has been previously published (28)]. For the final analysis, we included patients without TP53 abnormalities: 103 patients treated with G-CHL and 80 patients treated with IB (**Figure 1A**).

Patients’ characteristics are summarized in **Table 1**. Patients belonging to the two arms were balanced (i.e., *p*-values > 0.05) for age (74.7 years *vs*. 69.2 years), male gender (66% *vs*. 53%), advanced Rai stage (59% *vs*. 46%), increased β2-microglobulin levels (both 54%), and 11q22–23 deletion by FISH (11% *vs*. 16%). We observed that more patients treated with G-CHL were octogenarian (20% *vs*. 5%, *p* = 0.0038), were comorbid (median CIRS 6 *vs*. 4, *p* = 0.0009), and had an impaired kidney function (67% *vs*. 48%, *p* = 0.0061). In addition, a higher rate of U-IGHV patients received IB as frontline treatment compared to G-CHL (74% *vs*. 55%, *p* = 0.0087).

**Table 1:** Characteristics of recruited patients.

	G-CHL *n* = 103	IBRUTINIB *n* = 80	*p*-values
Age (median ± sd, years)	74.7 ± 6.6	69.2 ± 6.9	0.1064
≥80 years (%)	20 (20%)	4 (5%)	**0.0038**
Male/Female (%)	68 (66%)/35 (34%)	42 (53%)/38 (47%)	0.0935
Median CIRS (range)	6 (2-18)	4 (0-12)	**0.0009**
Median creatinine cl. ± sd (ml/min)	61.2 ± 17.5	66.7 ± 14.0	**0.0011**
Creatinine cl. < 70 ml/min (%)	69 (67%)	38 (48%)	**0.0061**
Rai stage III–IV (%)	62 (59%)	37 (46%)	0.0743
β2-microglobulin >3.5 mg/L (%)	53 (54%)	34 (54%)	>0.9999
IGHV status U/M (%)	56 (55%)/47 (45%)	59 (74%)/21 (26%)	**0.0087**
FISH del11q- (%)	11 (11%)	13 (16%)	0.5417
**Overall Response Rate (ORR)**	90 (87%)	69 (86%)	
CR	26 (25%)	5 (6%)	**0.0029**
PR/PR-L	64 (62%)	64 (80%)	
SD/PD	13 (13%)	11 (14%)	

CIRS, cumulative illness rating scale; creatinine cl., creatinine clearance; IGHV status U/M, unmutated/mutated; CR, complete remission; PR, partial remission; PR-L, partial remission with lymphocytosis; SD, stable disease; PD, progressive disease; sd, standard deviation. Bold values means statistically significant variables.

Eighty-three percent of patients received all the eight scheduled doses of G and chlorambucil; treatment was reduced or discontinued by 35% of patients. Forty-four percent of patients decreased the dose of IB and 79% were still under IB treatment at the last follow-up.

### Efficacy

After 9 months of treatments (i.e., 2–3 months after the end of the G-CHL), the overall response rate (ORR) according to iwCLL criteria was 87% for G-CHL and 86% for IB (**Figure 1B**). Despite a similar ORR, a higher rate of patients treated with G-CHL achieved a CR (**Table 1**, 25% *vs*. 6%, *p* = 0.0029) according to the iwCLL criteria (i.e., normalized complete blood count, negative CLL residue in the bone marrow, and lymph node size <1.5 cm). As expected, in the IB arm, there was a higher rate of PR/PR-L (**Table 1**, 62% *vs*. 80%). Variables associated with the achievement of a CR were M-IGHV (*p* = 0.0093), creatinine clearance (*p* = 0.0271), and G-CHL therapy (*p* = 0.006) (**Table S1**).

### Survival analysis

After a median follow-up of 30 months, 24 patients have relapsed in the G-CHL arm and 3 patients have relapsed in the IB arm; 17 patients required a subsequent treatment in the G-CHL arm (14 BTK inhibitors and 3 venetoclax ± rituximab) and 2 patients in the IB arm (both venetoclax ± rituximab); 10 patients died (4 due to sepsis, 2 due to pneumonia, 2 due to CLL, and 2 due to cardiovascular events) in the G-CHL arm *vs*. 8 in the IB arm (3 due to cardiovascular events, 1 due to RS, 1 due to pneumonia, 1 due to sepsis, 1 due to lung cancer, and 1 due to unknown cause). None developed a Richter syndrome transformation with G-CHL, but 1 did in the IB arm.

Overall, IB was associated with better PFS and TTNT but not with OS compared to G-CHL (**Figures 1C–E**). The 30-month PFS was 68% and 98%, and the estimated 5-year PFS was 61% and 82% for G-CHL and IB, respectively (*p* = 0.0061). Patients who received IB as frontline therapy had a 2.5-fold lower risk of disease progression or death than patients in the G-CHL arm (HR 2.58, 95% CI 1.38–4.84) (**Figure 1C**).

The 30-month TNTT was 88% and 97%, and the estimated 5-year TTNT was 61% *vs*. 97% for G-CHL and IB, respectively (*p* = 0.0043). IB was associated with a sixfold decrease in the need of a second line of treatment (HR 6.07, 95% CI 2.39–10.44) (**Figure 1D**).

The 30-month OS was 91% and 96% for G-CHL and IB, respectively (*p* = 0.6642), without a significant difference at 5 years (88% *vs*. 86%) (**Figure 1E**).

**Figure 2:**
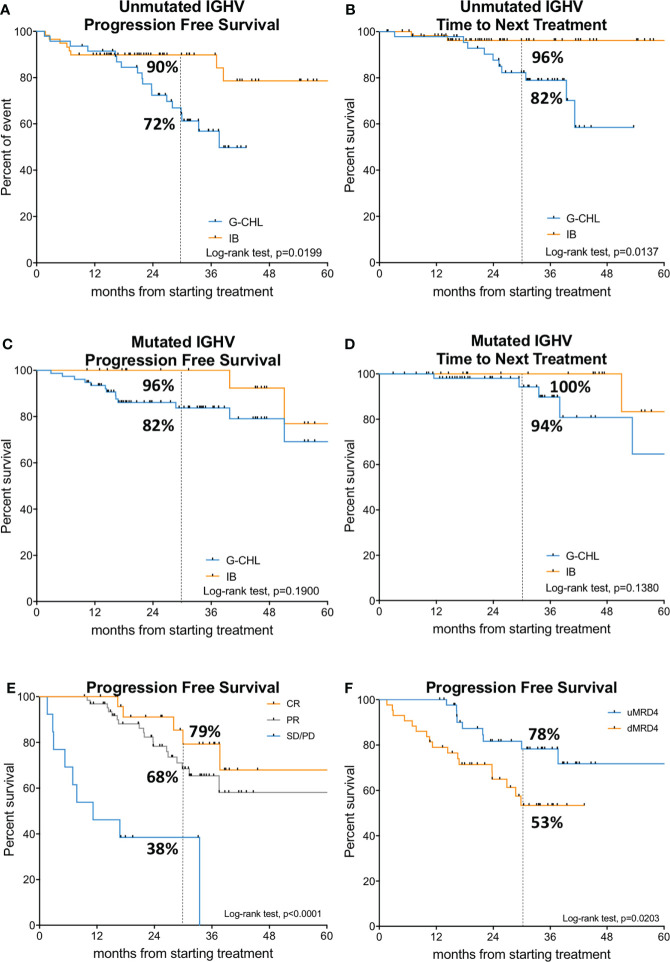
Survival analysis based on IGHV mutational status and deep of response. In the upper and middle panels, the Kaplan–Meier curves of progression-free survival and time to next treatment of IGHV unmutated (**A, B**); G-CHL, *n* = 56; IB, *n* = 59) and mutated patients (**C, D**); G-CHL, *n* = 47; IB, *n* = 21) are shown. In the lower panel, the Kaplan–Meier curves of progression-free survival of the G-CHL arm (*n* = 103) according to the iwCLL response rate on the left **(E)** and MRD (measurable residual disease) response on the right (*n* = 87) **(F)** are displayed.

Given that somatic hypermutation of the IGHV gene is one of the most important prognostic and predictive markers in CLL (24, 25, 29, 30), we assessed the impact of the IGHV mutational status in our patients. In U-IGHV patients, the 30-month PFS and TTNT were 72% *vs*. 90% (*p* = 0.0199, HR 2.58, 95% CI 1.19–5.57) and 82% *vs*. 96% (*p* = 0.0137, HR 5.38, 95% CI 1.73–11.69) for G-CHL and IB, respectively (**Figures 2A, B**). The median PFS was reached by G-CHL-treated U-IGHV patients at 37.7 months, while it was not reached by patients treated with IB. In M-IGHV patients, the 30-month PFS and TTNT were 82% *vs*. 96% (*p* = 0.1900, HR 2.54, 95% CI 0.83–7.84) and 94% *vs*. 100% (*p* = 0.1380, HR 3.93, 95% CI 0.93–13.64) for G-CHL and IB, respectively (**Figures 2C, D**).

### Impact of depth of response and MRD in the G-CHL arm

Subsequently, we analyzed the impact of depth of clinical response and MRD on the survival of patients in the G-CHL arm. According to iwCLL response rates, the median PFS was not reached for patients in PR and CR, but it was only 11.2 months for patients who did not respond to G-CHL therapy (i.e., classified as SD or PD) (*p* < 0.001). The 30-month PFS was 79%, 68%, and 38% for patients who achieved CR, PR, and SD/PD, respectively (**Figure 2E**). TTNT was not impacted by the type of response rate (**Figure S1A**). Conversely, patients with SD/PD had a shorter OS (median OS, 34.1 months), while it was superimposable for patients who achieved a CR or PR (30-month OS, 95.7% *vs*. 94.9% *vs*. 61.5%, *p* < 0.0001, **Figure S1B**).

Eighty-seven (75%) patients of the G-CHL arm were studied locally for MRD by flow cytometry in the peripheral blood. No patient with PD was studied for MRD. Considering all the 103 patients treated with G-CHL at disease evaluation (i.e., month +8 or +9), 43% of patients were able to achieve a uMRD in the peripheral blood, 43% had a dMRD, and 16% were not assessed (**Figure S1A**). Forty-nine patients were assessed for MRD in the bone marrow, 10% achieved uMRD, 38% had detectable MRD, and 52% were not studied. Ten patients (20%) had uMRD both in the peripheral blood and in the bone marrow, 8 (16%) had uMRD in the peripheral blood but a dMRD in the bone marrow, and 31 (63%) had a dMRD both in the peripheral blood and in the bone marrow. The concordance rate between peripheral blood and bone marrow assessment was 83%. Variables associated with uMRD in the peripheral blood were an M-IGHV status (*p* = 0.0219) and creatinine clearance (*p* = 0.0311).

The 30-month PFS was significantly higher for patients achieving uMRD4, which was 78% *vs*. 53% for uMRD patients and dMRD patients, respectively (*p* = 0.0203) (**Figure 2F**). The median PFS was not reached. Patients with dMRD at the end of the G-CHL treatment had a 2.5-fold greater risk of progression than those with uMRD (HR 2.49, 95% CI 1.15–5.43).

TTNT was also influenced by the MRD response with an estimated median TTNT of 43.2 months for patients with dMRD, while it was not reached for those with uMRD (**Figure S2D**). The 30-month TTNT was 96.7% *vs*. 74.2% for uMRD and dMRD patients (*p* = 0.0211) (**Figure S1G**). Patients with dMRD were at threefold greater risk of starting a new treatment than those with uMRD (HR 3.4, 95% CI 1.19–9.92).

### Adjusted and propensity score matched analysis

An unadjusted Cox analysis performed joining all the patients of both arms (*n* = 183 patients) showed that IB was significantly more effective than G-CHL in decreasing the risk of disease progression (HR 0.37, *p* = 0.0078) or next line of therapy (HR 0.14, *p* = 0.0086) in treatment-naïve patients with CLL (**Table S2**). To minimize the confounding effect, we adjusted the relationship between treatment arms (IB *vs*. G-CHL), PFS, and TTNT for all the variables skewed between arms (**Table 1**), as well as for all variables significantly associated with PFS and TTNT in the Cox univariate analysis (**Table S2**). After introducing these covariates into a multiple Cox regression model, the protective effect of IB *vs*. G-CHL in terms of risk of disease progression (HR 0.32, 95% CI 0.13–0.81, *p* = 0.0163) or next treatment (HR 0.12, 95% CI 0.03–0.61, *p* = 0.0102) was confirmed independently of potential confounders (**Table S3**).

Given the relevant differences of comorbidities and IGHV status between G-CHL and IB arms, we also performed a propensity score matched analysis (1:1). New arms were created, either with (*n* = 79) or without (*n* = 50) replacement balancing differences among treatment groups (**Tables S4**, **S5**). Even after this matched analysis, PFS and TTNT, but not OS, were longer in the IB arm than in the G-CHL arm (**Figures S2A, B**).

### Safety and economic analysis

Overall, patients treated with G-CHL had more AEs than those receiving IB (2.98 *vs*. 1.68 AE/month of treatment/person), and less ambulatory outpatient visits (RR 0.17, 95% CI 0.15–0.20) and hospitalizations (RR 0.42, 95% CI 0.17–1.10). However, only the number of outpatient visits was statistically significant.

Ninety-eight percent of patients received premedication (paracetamol 1 g iv, anti-H1 iv, and methyl-prednisolone iv) before G infusion. Infusion-related reactions (IRRs) were recorded in 36.9% of patients, the majority being grade 1 or grade 2 and only 4.9% being grade 3. Given the retrospective nature of the study, we focus only on severe (grade ≥3) AEs. The most relevant G ≥ 3 AEs were neutropenia (35% *vs*. 9%, *p* < 0.0001), infections (13% *vs*. 16%, *p* = 0.3188), thrombocytopenia (12% *vs*. 1%, *p* = 0.0004), anemia (6% *vs*. 0%, *p* = 0.0002), and atrial fibrillation (2% *vs*. 9%, *p* = 0.0813) for G-CHL and IB, respectively. No tumor lysis syndrome occurred.

An economic analysis was carried out on 92 patients, 69 patients from the G-CHL arm and 23 patients from the IB arm. The characteristics of the economic cohort is reported in **Table S6**. As shown in **Figure S2C**, IB was associated with higher monthly costs, mainly related to the costs of the drug rather than the management of AEs. The mean total monthly cost per patient was €1,545 with G-CHL and €5,587 with IB, resulting in a mean savings per month of €4,074 (95% CI 3,267–4,881). This difference is mainly due to the savings in first-line drug cost (€1,029 *vs*. €5,297) and slightly to the decrease in hospitalization and/or outpatient visits (€95 *vs*. €290) (**Figure S2C**).

## Discussion

We gathered data from 183 CLL patients without TP53 abnormalities who were treated with continuous IB or with 6 months of G-CHL therapy as first-line therapy in the real-life setting. We found that (i) a remarkable number of patients were able to achieve a uMRD with G-CHL, and (ii) PFS and TTNT, but not OS, were better with IB than with G-CHL. The similar OS is likely due to the fact that all patients received targeted therapies with either a BTK or a BCL2 inhibitor as second-line therapy.

Furthermore, recent studies found that a high number of comorbidities, assessed by the CIRS score, have a detrimental impact of target therapies’ efficacy (31–33). In our study, despite a relevant number of comorbid patients, they showed a remarkable outcome with G-CHL.

The IGHV mutational status is one of the most important prognostic and predicted markers in CLL, being able to identify patients who might benefit most from a fixed-duration therapy (6, 10, 30, 34). When PFS and TTNT curves were stratified for the IGHV status, we found that IB improvement was significant only for the U-IGHV patients. Conversely, among M-IGHV patients after a median follow-up of 30 months, the PFS and TTNT curves of the G-CHL and IB almost overlapped, thus suggesting that fixed-duration therapy might be a key strategy in M-IGHV CLL patients in clinical practice.

G-CHL treatment was approved based on the results of the CLL11 trial, where G-CHL was compared with rituximab-CHL and CHL alone (35). The median age was 73 years (range, 39–90 years); 61% were U-IGHV, 8% harbored a del17p-, and 16% harbored a del11q-. All patients had a CIRS score >6 and/or a creatinine clearance <70 ml/min. G-CHL led to a better PFS, TTNT, and OS than the other arms. A uMRD in the peripheral blood at the end of treatment was significantly more common in patients receiving G-CHL compared to those who received rituximab-CHL (35.8% *vs*. 3.3%, *p* < 0.001). Patients with uMRD had a median PFS of 56.4 months compared to 23.9 months for patients categorized as MRD intermediate (MRD events between 10^−4^ and 10^−2^) and 13.9 months for dMRD patients (*p* < 0.001). MRD response was also significantly associated with a better OS (35). In our study data, we excluded patients with TP53 abnormalities (deletion or mutation) and fewer patients harbored U-IGHV and/or del11q- by FISH. The presence of fewer patients with unfavorable markers in our study might explain the higher uMRD rate (43% *vs*. 35.8%) and the longer PFS. Furthermore, G premedication significantly decreased IRR (66% in the CLL11 trial *vs*. 36.9% in our study, G3 21% in the CLL1 trial *vs*. 4.9% in our study).

Since CHL is a weak partner, G has been combined with continuous IB [iLLUMINATE trial (36)], continuous acalabrutinib [ELEVATE TN (37)], or the 12-month venetoclax [CLL14 (10)] and compared with G-CHL. In all these trials, the combination of G plus an oral targeted drug led to higher uMRD rates, particularly for G-venetoclax, and sustained longer PFS than G-CHL. Remarkably, IRRs were lower when G was given in combination with BTK inhibitors (36, 37).

Recently, G-CHL has been compared with the fixed-duration oral therapy IB-venetoclax (38). The GLOW trial included patients ≥65 years old or those with CIRS score ≥6 or creatinine clearance <70 ml/min. The uMRD rate in the bone marrow by next-generation sequencing was significantly higher for IB-venetoclax than for G-CHL (56% *vs*. 21%, *p* < 0.001), which led to a significantly longer PFS. The improvement in PFS with IB-venetoclax was consistent across patients ≥65 years and/or with a CIRS ≥ 6.

A041202 is a phase 3 clinical trial comparing IB ± rituximab with another chemoimmunotherapy schedule used in elderly patients, i.e., bendamustine-rituximab (BR) (39). With a median follow-up of 55 months, the median PFS was 44 months with BR and was not reached in the IB arms. An economic analysis showed that costs (associated with protocol-specified resource use) were significantly higher for patients receiving IB ± rituximab (mean $189,335 or $219,908; *p* < 0.0001) compared to BR (mean $51,345), driven by the higher costs for IB (40). Quality-adjusted life years were also similar between arms. In line with our data, IB provides better disease control in patients with del11q by FISH and U-IGHV, counteracted by a much higher cost of the drug. IB plus rituximab was also tested against FCR in CLL patients aged ≤70 years in the E1912 trial (41). With a median follow-up of 5.8 years, the median PFS was superior for IB-rituximab (*p* < 0.001). Notably, only in the E1912 trial did IB-rituximab improve not only PFS compared to FCR in patients with IGHV mutated and unmutated gene (HR 0.27, *p* < 0.001) but also OS (HR 0.47, *p* = 0.018).

The main limitation of our study is its retrospective structure and the sample size. To minimize selection and attrition biases as well as imprecise reporting of data inherent to observational studies, we asked the treating physician to report all CLL patients treated frontline with G-CHL. We analyzed the reported data, excluded cases with TP53 abnormalities, and performed computerized manual consistency checks on each case report form. Furthermore, given the differences in the clinical characteristics of patients (**Table 1**), particularly age and comorbidities, we applied a propensity score matched analysis with (*n* = 79) and without (*n* = 50) replacement balancing (**Tables S4**, **S5**). The small size of the samples affects the conclusions of the study. In addition, the median follow-up of 30 months does not allow us to reach conclusions about the OS.

The Italian CLL campus experience with G-CHL confirms the effectiveness of this treatment, particularly for M-IGHV patients capable of reaching a CR or a uMRD. Although MRD assessment is still not recommended by current guidelines, an increasing number of centers utilize this analysis (42). Continuous treatment with IB provides longer remission in elderly CLL patients unfit for fludarabine-based therapy (31). However, it is noteworthy that some patients can achieve long-term disease control with a less expensive fixed-duration obinutuzumab-based therapy, which may represent an option for first-line treatment in countries with economic constraints (8, 9).

## Data availability statement

The raw data supporting the conclusions of this article will be made available by the authors, without undue reservation.

## Ethics statement

The studies involving human participants were reviewed and approved by Azienda Ospedale Università Paadova. The patients/participants provided their written informed consent to participate in this study.

## Author contributions

AV designed the study, performed statistical analysis, visited patients, and wrote the article. GC, AF, CV, AS, FC, PS, MG, GR, FQ, VM, AG, MM, LS, GR, SP, FA, and ACe provided intellectual inputs and visited patients. FM, ACu, RF, SM, MC, LL, PG, and LT visited patients, provided intellectual inputs, and reviewed the article. All authors contributed to the article and approved the submitted version.

## Funding

This work was supported by funds to LT from Associazione Italiana per la Ricerca sul Cancro (A.I.R.C.) projects (IG-25024), “Ricerca per Credere nella Vita” RCV odv to SP, and Roche spa to LT. Roche sponsored a fellowship to the University of Padova for collecting and analyzing the data, which was won by SP.

## Conflict of interest

AV received honoraria from Janssen, Abbvie, CSL Behring, and Italfarmaco. LT received research funding from Gilead, Roche, Janssen, and Takeda, and is on the advisory board for Roche, Takeda, Abbvie, and AstraZeneca. GR received research funding from Gilead. FM is on the advisory board for Janssen, Takeda, and Abbvie. ACu is on the advisory board and speaker bureau for Roche, Abbvie, Gilead, and Janssen. RF is on the advisory board or speaker bureau for Incyte, Amgen, AstraZeneca, Janssen, Gilead, and Novartis. LL received honoraria from Abbvie, Janssen, Astra Zeneca, and Beigene. FQ plays an advisor role for AstraZeneca and Janssen, is a speaker for Janssen, and is a consultant for Sandoz. LS received honoraria from AbbVie, AstraZeneca, and Janssen.

The remaining authors declare that the research was conducted in the absence of any commercial or financial relationships that could be construed as a potential conflict of interest.

## Publisher’s note

All claims expressed in this article are solely those of the authors and do not necessarily represent those of their affiliated organizations, or those of the publisher, the editors and the reviewers. Any product that may be evaluated in this article, or claim that may be made by its manufacturer, is not guaranteed or endorsed by the publisher.
