# Supplementary material for: Obinutuzumab plus chlorambucil versus ibrutinib in previously untreated chronic lymphocytic leukemia patients without TP53 disruptions: A real-life CLL campus study
Source: Front Oncol. 2022 Nov 21;12:1033413. doi: 10.3389/fonc.2022.1033413 (PMC9719965; doi:10.3389/fonc.2022.1033413)
Supplement: Supplementary file 1 [file DataSheet_1.docx]

**SUPPLEMENTARY METHODS**

*IGHV mutational status*

Analysis of the IGHV mutational status was performed within 12 months from diagnosis on peripheral blood CLL cells from fresh samples or frozen purified CLL cells harvested in DMSO. RNA was extracted from 2x106 B cells using the RNeasy™ Total RNA kit (Qiagen, Hilgen, Germany) and reverse transcribed using the SuperScript™ Preampliﬁcation System for ﬁrst-strand cDNA synthesis (Life Technologies, Carlsbad, CA). The CLL B-cell HV gene family was assigned as previously described(25, 26). HV gene sequences were determined by amplifying 5μl of the original cDNA using the appropriate HV leader and HC primers. PCR products were directly sequenced after puriﬁcation with the Wizard PCR Preps (Promega, Madison, WI) using an automated genetic analyzer (3130 ABI Applied Biosystems, Foster City, CA, USA). Sequences were analyzed using the IMGT/VQUEST and BLAST softwares(27) to detect VDJ junction. Cases with a sequence homology <98 from the corresponding germline gene were considered as mutated (M-IGHV), and those with a homology ≥98% as unmutated (U-IGHV)(28, 29). Stereotyped B-cell receptor (BCR) was assessed with ARResT(30, 31).

*Cytogenetics by fluorescence in situ hybridization (FISH) and mutations*

FISH was performed on standard cytogenetic preparations from peripheral blood(26). The slides were hybridized with the multicolor probe set LSI p53/LSI ATM and LSI D13S319/LSI 13q34/ CEP12 and RP11-177O8 according to the manufacturer’s instructions(32). Three hundred interphase nuclei were analyzed for each probe and the cut-off for positive value was 10% for deletion of 11q22.3 (ATM), 17p13.1 (TP53) loci and 13q14.3 (D13S319), and 5% for trisomy 12. TP53 gene sequencing was performed according to ERIC guideline assessing exons 4-10; if negative exons 2, 3 and 11 were also investigated(22).

**SUPPLMENTARY TABLES**

Table S1. Variables associated with response rates.

|  | **CR**  n = 31 | **PR/PR-L/SD/PD**  n = 152 | **p values** |
| --- | --- | --- | --- |
| ≥ 75 years | 16 (51.6%) | 83 (54.6%) | 0.5582 |
| Male | 16 (51.6%) | 98 (64.5%) | 0.2227 |
| CIRS > 6 | 15 (48.4%) | 67 (44.1%) | 0.6953 |
| Creatinine Clearance < 70ml/min | 24 (77.4%) | 84 (55.3%) | **0.0271** |
| Rai stage III-IV | 16 (51.6%) | 82 (53.9%) | 0.8453 |
| Mutated IGHV | 21 (67.7%) | 56 (36.8%) | **0.0093** |
| FISH del11q- | 3 (9.7%) | 13 (8.6%) | 0.9999 |
| Dose reduction | 11 (35.5%) | 56 (36.8%) | 0.9999 |
| G-GHL arm | 26 (83.9%) | 77 (50.7%) | **0.0006** |

Table S2. Univariate COX model for progression free survival and time to next treatment

|  | **Progression Free Survival** | | | **Time to Next Treatment** | | |
| --- | --- | --- | --- | --- | --- | --- |
|  | **HR** | **95% C.I.** | **p values** | **HR** | **95% C.I.** | **p values** |
| ≥ 80 years | 3.36 | 1.71-6.58 | **0.0004** | 1.79 | 0.50-6.38 | 0.3700 |
| Male | 0.94 | 0.49-1.79 | 0.8470 | 1.26 | 0.47-3.36 | 0.6460 |
| CIRS > 6 | 1.78 | 0.94-3.53 | 0.0749 | 1.67 | 0.65-4.24 | 0.2840 |
| Cr. Clear. < 70 ml/min | 0.61 | 0.84-3.10 | 0.1440 | 1.41 | 0.55-3.64 | 0.4730 |
| Rai stage III-IV | 1.12 | 0.59-2.11 | 0.7260 | 1.49 | 0.58-3.81 | 0.4090 |
| Unmutated IGHV | 1.86 | 1.02-3.52 | **0.0494** | 1.79 | 0.66-4.87 | 0.2520 |
| FISH del11q- | 1.82 | 0.83-3.97 | 0.1340 | 2.83 | 0.89-7.09 | 0.0572 |
| Ibrutinib arm | 0.37 | 0.17-0.76 | **0.0078** | 0.14 | 0.03-0.60 | **0.0086** |

Table S3. Multivariate COX model for progression free survival and time to next treatment

|  | **Progression Free Survival** | | | **Time to Next Treatment** | | |
| --- | --- | --- | --- | --- | --- | --- |
|  | **HR** | **95% C.I.** | **p values** | **HR** | **95% C.I.** | **p values** |
| ≥ 80 years | 2.87 | 1.36-6.05 | **0.0056** | 1.44 | 0.37-5.50 | 0.5977 |
| CIRS > 6 | 1.58 | 0.74-3.37 | 0.2356 | 1.18 | 0.36-3.84 | 0.7847 |
| Cr. Clear. < 70 ml/min | 1.12 | 0.53-2.39 | 0.7650 | 0.74 | 0.24-2.30 | 0.6055 |
| U-IGHV | 2.21 | 0.77-6.31 | 0.1395 | 1.09 | 0.22-5.34 | 0.9184 |
| Ibrutinib arm | 0.32 | 0.13-0.81 | **0.0163** | 0.13 | 0.03-0.61 | **0.0102** |

Table S4. New G-CHL and IB cohorts after propensity score matched without replacement.

|  | **Post PSM without replacement** | | |
| --- | --- | --- | --- |
|  | **G-CHL (N=50)** | **IB (N=50)** | **p-value** |
| Age, years (range) | 74 (70-78) | 74 (70-78) | 0.8794 |
| Female | 18 (36%) | 19 (39%) | 0.6807 |
| Duration of disease (years) | 3.8 (1.6-7.4) | 3.3 (1.3-6.4) | 0.5532 |
| CIRS(%) | 4 (3-6) | 4,5 (4-7) | 0.0702 |
| RAI Stage(%) |  |  | 0.1054 |
| III | 24 (48%) | 17 (34%) |  |
| IV | 5 (10%) | 13 (26%) |  |
| Creatinine Clearance (ml/min) | 56 (48 to 67) | 63 (52 to 74) | 0.0804 |
| Unmutated IGHV (%) | 21 (54%) | 22 (44%) | 0.3977 |
| FISH del11q- (%) | 6 (12%) | 5 (10%) | >0.9999 |

Table S5. New G-CHL and IB cohorts after propensity score matched with replacement.

|  | **Post PSM with replacement** | | |
| --- | --- | --- | --- |
|  | **G-CHL (N=79)** | **IB (N=79)** | **p-value** |
| Age, years (range) | 75 (70-79) | 74 (71-79) | 0.6304 |
| Female (%) | 25 (32%) | 21 (27%) | 0.6050 |
| Duration of disease (years) | 4.1 (2.3-7.0) | 3.8 (1.8-8.0) | 0.7894 |
| Median CIRS | 6.5 (4-8.8) | 6 (4-7) | 0.5395 |
| RAI Stage (%) |  |  | 0.1007 |
| III | 26 (33%) | 29 (36%) |  |
| IV | 22 (28%) | 11 (14%) |  |
| Creatinine clearance (ml/min) | 59 (49-71) | 60 (49-72) | 0.9217 |
| Unmutated IGHV (%) | 39 (49%) | 32 (40%) | 0.3212 |
| FISH del11q- (%) | 8 (10%) | 6 (8%) | 0.7807 |

Table S6. Clinical characteristic of the 82 patients of the economic cohort.

|  | ECONOMIC COHORT | | |  |
| --- | --- | --- | --- | --- |
|  | **G-CHL (N=69)** | **IB (N=23)** | **p-value** | |
| Age, years (range) | 76 (72-82) | 734 (71-78) | 0,1135 | |
| Female (%) | 21 (30%) | 8 (35%) | 0,7966 | |
| Duration of disease (years) | 4.2 (2.4-7.4) | 3.8 (1.9-6.3) | 0,7048 | |
| Median CIRS | 7 (4-9) | 6 (4.5-8.0) | 0,7683 | |
| Stage |  |  | 0,0003 | |
| III | 23 (33%) | 7 (30%) |  | |
| IV | 24 (35%) | 2 (9%) |  | |
| Creatinine clearance (ml/min) | 57 (47-71) | 67 (61-71) | 0,0573 | |
| Unmutated IGHV (%) | 29 (51%) | 15 (65%) | 0,3224 | |
| FISH del11q (%) | 8 (12%) | 4 (17%) | 0,4863 | |

**LEGENDS TO SUPPLEMENTARY FIGURES**

**Figure S1 Impact of response rate and MRD rate in G-CHL patients.** In the upper panel there are Kaplan Meier curves of time to next treatment (A) and overall survival (B) according to the iwCLL response rate of patients treated with G-CHL. In the middle panel (C) there is the histograms of MRD rate in the peripheral blood and bone marrow. In the lower panel there are Kaplan Meier curves of time to next treatment (D) and overall survival (E) according to MRD of patients treated with G-CHL.

**Figure S1. Propensity score match and economic analysis.** In the upper panel (A) there are Kaplan Meier curves of progression free survival, time to next treatment and overall survival after propensity score match analysis without resampling. In the middle panel (B) there are Kaplan Meier curves of progression free survival, time to next treatment and overall survival after propensity score match analysis with resampling. In the lower panel (C) there is histogram of economic costs of G-CHL, IB and their difference.
